# Associations of Co-occurring Symptom Trajectories With Sex, Race, Ethnicity, and Health Care Utilization in Children

**DOI:** 10.1001/jamanetworkopen.2023.14135

**Published:** 2023-05-18

**Authors:** Terri Voepel-Lewis, Thea Senger-Carpenter, Bingxin Chen, Julia Seng, Cherie Cofield, Robert Ploutz-Snyder, Eric L. Scott

**Affiliations:** 1 Department of Anesthesiology at Michigan Medicine, University of Michigan, Ann Arbor; 2 School of Nursing, University of Michigan, Ann Arbor; 3 Department of Pediatrics at Michigan Medicine, University of Michigan, Ann Arbor

## Abstract

**Importance:**

Co-occurring physical and psychological symptoms during childhood and early adolescence may increase risk of symptom persistence into adulthood.

**Objective:**

To describe co-occurring pain, psychological, and sleep disturbance symptom (pain-PSS) trajectories in a diverse cohort of children and the association of symptom trajectory with health care utilization.

**Design, Setting, and Participants:**

This cohort study was a secondary analysis of longitudinal data from the Adolescent Brain Cognitive Development (ABCD) Study, collected between 2016 and 2022 at 21 research sites across the US. Participants included children with 2 to 4 complete annual symptom assessments. Data were analyzed from November 2022 to March 2023.

**Main Outcomes and Measures:**

Four-year symptom trajectories were derived from multivariate latent growth curve analyses. Pain-PSS scores, including depression and anxiety, were measured using subscales from the Child Behavior Checklist and the Sleep Disturbance Scale of Childhood. Nonroutine medical care and mental health care utilization were measured using medical history and *Diagnostic and Statistical Manual of Mental Disorders* (*Fifth Edition*) items.

**Results:**

A total of 11 473 children (6018 [52.5%] male; mean [SD] age at baseline, 9.91 [0.63] years) were included in analyses. Four no pain-PSS and 5 pain-PSS trajectories were supported with good or excellent model fit (predicted probabilities, 0.87-0.96). Most children (9327 [81.3%]) had asymptomatic or low, intermittent, or single symptom trajectories. Approximately 1 in 5 children (2146 [18.7%]) had moderate to high co-occurring symptom trajectories that persisted or worsened. Compared with White children, there was a lower relative risk of having moderate to high co-occurring symptom trajectories among Black children (adjusted relative risk ratio [aRRR] range, 0.15-0.38), Hispanic children (aRRR range, 0.58-0.67), and children who identified as another race (including American Indian, Asian, Native Hawaiian, and other Pacific Islader; aRRR range, 0.43-0.59). Less than half of children with moderate to high co-occurring symptom trajectories used nonroutine health care, despite higher utilization compared with asymptomatic children (nonroutine medical care: adjusted odds ratio [aOR], 2.43 [95% CI, 1.97-2.99]; mental health services: aOR, 26.84 [95% CI, 17.89-40.29]). Black children were less likely to report nonroutine medical care (aOR, 0.61 [95% CI, 0.52-0.71]) or mental health care (aOR, 0.68 [95% CI, 0.54-0.87]) than White children, while Hispanic children were less likely to have used mental health care (aOR, 0.59 [95% CI, 0.47-0.73]) than non-Hispanic children. Lower household income was associated with lower odds of nonroutine medical care (aOR, 0.87 [95% CI, 0.77-0.99]) but not mental health care.

**Conclusions and Relevance:**

These findings suggest there is a need for innovative and equitable intervention approaches to decrease the potential for symptom persistence during adolescence.

## Key Points

**Question:**

Are co-occurring pain, psychological, and sleep disturbance symptom trajectories associated with demographic characteristics of children and are symptom trajectories associated with nonroutine health care utilization during early adolescence?

**Findings:**

This cohort study, including 11 473 children, used 9 symptom trajectories to classify participants in the Adolescent Brain Cognitive Development (ABCD) study cohort, most of whom clustered with asymptomatic or low, intermittent, or single symptom trajectories. Approximately 1 in 5 children had moderate to high co-occurring symptom trajectories, but less than half of these reported nonroutine medical or mental health care use, with lower rates among Black, other race, and Hispanic children.

**Meaning:**

These findings suggest that there is a need for better recognition and equitable, prompt interventions to mitigate symptom persistence among children.

## Teaser

This cohort study assesses co-occurring pain, psychological, and sleep disturbance symptom trajectories in a diverse cohort of children and the association of symptom trajectory with health care utilization

## Introduction

Understanding symptom risk during childhood and adolescence is essential, since somatic and psychological symptoms have recently worsened for many youths,^1,2,3^ with a 43% increase in mental health–related emergency department visits from 2015 to 2022.^4^ Although pain, anxiety, or depression symptoms are self-limited for most children,^5,6,7^ they can persist throughout adolescence and into adulthood for many.^8,9^ Indeed, 1 in 3 adolescents report persistent pain,^10^ and 1 in 5 adolescents report lingering depression or anxiety.^11,12^ Sleep disturbance accompanies persistent symptoms for as many as 17% of youths,^13,14,15^ compounding the symptom burden and its effects on academic, social, and emotional development.^16^ Such high rates of symptom persistence suggest that pain and mental health symptoms remain poorly recognized or addressed for many youths.

Co-occurrence of pain, psychological, and sleep disturbance symptoms (pain-PSS) may reflect risk of symptom persistence and negative health outcomes. However, disentangling the emergence of co-occurring symptoms remains elusive, since most studies describe single symptom trajectories across developmental age groups or in clinical samples. For instance, higher anxiety symptoms, but not depression symptoms, were associated with increasing pain trajectories in economically deprived youths aged 2 to 16 years.^17^ Anxiety symptoms were also associated with sporadic, frequent pain trajectories in a Canadian cohort.^18^ Depression symptoms at age 16 years were associated with persistent and severe musculoskeletal pain trajectories at age 43 years in another cohort.^19^ Furthermore, anxiety and depression symptoms were both associated with high pain impairment trajectories in children and youths aged 6 to 18 years with chronic abdominal pain.^20^

These data provide insight into symptom risk during adolescence, yet a better understanding of phenotypic symptom profiles is warranted, since pain-PSS may emerge simultaneously. This is particularly germane, since co-occurring symptoms may influence and exacerbate one another, contributing to their mutual maintenance and eventual chronicity.^21,22^ Indeed, we recently found that children with comorbid pain-PSS were 5 times more likely than those with no or low symptoms to have persistent or recurrent pain the following year.^23^ Given the high costs of treating chronic pain (estimated at >$19.5 billion annually)^24^ and comorbid mental health symptoms (additional $8.8 billion annually),^25^ early recognition and intervention is essential.

This study tested the hypotheses that co-occurring pain-PSS trajectories could be identified and differentiated in boys and girls by age 13 years, and that high co-occurring pain-PSS trajectories would be associated with greater health care utilization (HCU). We also examined the associations between child sex, race, ethnicity, trajectories, and HCU.

## Methods

This cohort study was deemed exempt from review and informed consent by the University of Michigan institutional review board because we used preexisting data from the Adolescent Brain Cognitive Development Study (ABCD release 4.0), with permission from the National Institute of Mental Health. The ABCD Study is a longitudinal study of adolescent health and development that used stratified probability sampling to recruit a sociodemographically diverse, representative cohort from 21 sites across the US.^26,27,28^ Described in detail elsewhere,^27^ the sampling plan included strategies to minimize systematic biases, with the understanding that self-selection was unavoidable.^27^ Comprehensive baseline assessments were completed by 6185 boys and 5681 girls aged 9 to 10 years and attrition, to date, has been minimal (0.01% withdrew; 4% late or missing visits).^29^ We included data from participants who completed 2 or more of the annual symptom assessments conducted between 2016 (baseline) and 2022 (most recent follow-up). This study is reported following the Strengthening the Reporting of Observational Studies in Epidemiology (STROBE) reporting guideline.

### Outcome Measures

#### Co-occurring Pain-PSS Trajectory

Given previously described, variable associations across childhood,^13,17,18^ we included measures of pain, anxiety, depression, and sleep disturbance symptoms to characterize our comorbid pain-PSS trajectories. Annual symptom assessments were derived from the parent-reported Child Behavior Checklist (CBCL)^30^ and Sleep Disturbance Scale for Children (SDSC).^31^ The CBCL is a valid, reliable survey widely used to screen for presence and severity of psychological and somatic symptoms.^30,32^ The SDSC includes valid and reliable subscales assessing sleep behaviors and symptoms.^31^

#### Pain Index

A sum of 3 CBCL items, each scored 0 (never) to 2 (often), measured past 6-month presence and frequency of abdominal pain, headache and general aches, and pains (range, 0, none, to 6, frequent multisite pain). Construct validity of the items was supported in children with persistent pain conditions.^33^

#### Anxiety Symptoms

Anxiety symptoms were assessed using raw scores from the 13-item CBCL Anxious/Depressed subscale. The CBCL Anxious/Depressed subscale ranges from 0 to 26, with higher scores indicating greater symptoms of apprehension and nervousness (eg, nervous or tense, cries a lot, fearful or anxious).

#### Depression

Depression was measured using the 8-item CBCL Withdrawn/Depressed subscale measured depression symptoms characterized by sadness and loss of interest (eg, unhappy, sad, depressed; withdrawn; shows little interest). Scores range from 0 to 16, with higher scores indicating worse depression symptoms.

#### Sleep Disturbance Symptoms

Sleep disturbance was measured using the 5-item SDSC somnolence subscale, with scores ranging from 0 to 20 and higher score indicating more sleep disturbance. Internal consistency was supported in the ABCD cohort (Cronbach α = .73).^23^

#### Health Care Utilization

Affirmative parent responses to either question, “During the past year has your child seen a doctor, nurse, dentist, or any other health professional other than for regular checkups?” or “Has he/she been to an emergency room in the past year?” were used to assess nonroutine medical care in the final year. The *Diagnostic and Statistical Manual of Mental Disorders* (*Fifth Edition*) (*DSM-5*) diagnostic survey item inquiring whether the child had received mental health or substance use services in the past year as our measure of mental HCU.

### Covariate Measures

#### Demographics

Annual parent report of age and baseline documentation of sex, race, Hispanic ethnicity, and household income were recorded. Race and ethnicity were categorized as Black, multiracial, White, and other (including American Indian, Asian, Native Hawaiian, and other Pacific Islander). Race and ethnicity characteristics were included in the analyses to describe potential differences in symptom trajectories and HCU. Annual household income was recoded as less than $35 000 or $35 000 or more to approximate the US poverty line split.

#### Pubertal Stage

We recorded the child’s self-reported puberty stage (from 1, indicating earliest, to 5, latest), which correlates with hormonal data in the cohort.^34^ We substituted 77 missing scores (1.3%) for boys and 306 missing scores (5.6%) for girls with parent-report.

#### Medical History Measures

We summed parent-reported child medical conditions (eg, asthma, diabetes) and injuries (eg, sprains, fractures) to derive a baseline comorbidity burden index (range, 0-26; higher score indicates more comorbidities).^35,36^ We also report baseline CBCL somatic symptoms subscale, minus the 3 pain items (range, 0-16; higher score indicates more somatic symptoms), and inattention symptom scores (range, 0-22; higher score indicates more inattention symptoms).

#### Self-reported Pain

We also report the child’s self-reported pain location as single region vs multiregion (≥2 body regions; eg, head and neck, abdomen and pelvis, chest, back).^37,38^ Children also self-reported their past-month pain intensity (0, no pain, to 10, worst pain) and pain interference with normal activities (0, not at all, to 10, stopped me from doing anything).

### Statistical Analysis

Following extensive preliminary modeling, we split the sample into no pain (ie, children who never complained of pain) and pain (ie, children who had pain on ≥1 assessment) strata, given that 35% of the cohort reported no pain across years. To test our first hypothesis, we used the Stata command traj to conduct person-centered, multivariable trajectory modeling for each strata, simultaneously fitting group-based latent class growth analyses for repeated observations of symptoms.^39^ We assumed a zero-inflated Poisson distribution for each symptom and used a polynomial relationship of order up to 3 to model the association between time and outcome. We estimated models with 3 to 6 groups based on past single symptom trajectory reports.^5,6,10,18^ For each group and outcome, we chose the highest order of polynomial that was statistically significant at *P* < .05. Final solutions were chosen based on Bayesian Information Criterion, proportional group sizes (with no group <5% of the sample), and, given numerous significant classes, parsimony and interpretability of the clusters.^40^ This analysis did not account for the nesting effect of site or family. We report group characteristics as frequency (%) or mean and SD, and used mixed-effect multinomial regression to examine fixed effects of baseline characteristics (sex, race, ethnicity) across all groups (no pain and pain strata included), controlling for pubertal stage, comorbidity index, somatic and inattention symptoms, and the random effects of family and research site. Adjusted relative risk ratios (aRRRs) with 95% CIs are reported, with the most asymptomatic group (ie, no pain and no PSS) as base comparator.

We used mixed-effect logistic regression models to examine the hypothesized association of symptom trajectory with HCU outcomes, adjusting for confounding effects of baseline characteristics (sex, puberty, race, ethnicity, comorbidity, household income). Models were weighted to account for the child’s posterior probability of belonging to their assigned cluster and effects of family and research site were included as random intercepts. These analyses were accomplished with Stata version 17 (StataCorp), with outcomes expressed as adjusted odds ratios (aORs) with 95% CIs. Missing data were not imputed or replaced. *P* values were 2-tailed, and statistical significance was set at *P* < .05. Data were analyzed from November 2022 to March 2023.

## Results

Analyses included 11 473 children (6018 [52.5%] male). Most children (10 293 [89.7%]) completed 3 to 4 of the annual symptom assessments, and 6169 final assessments (53.8%) were made in the third follow-up year. At baseline, the mean (SD) participant age was 9.91 (0.63) years, and children were relatively healthy (9306 [81.1%] with 0-1 medical conditions); mean (SD) age was 10.92 (0.64) years at year 1, 12.00 (0.66) years at year 2, and 12.90 (0.64) years at year 3. Nearly one-third of participants (3500 [30.5%]) had reached late puberty (stages 4-5) by their final assessment. Most children (7442 children [64.9%]) reported pain at 1 or more assessments (pain stratum), while 4031 children (35.1%) never reported pain (no pain stratum).

Trajectory analyses supported our first hypothesis with excellent model fit for selected solutions (ie, mean predicted probabilities of 0.87-0.96 supporting 4 no pain-PSS and 5 pain-PSS trajectories) (eTable 1 in Supplement 1). Most children (9327 children [81.3%]) had asymptomatic (no pain and no PSS) or low, intermittent, or single symptom trajectories (eFigure in Supplement 1). However, 2146 children (18.7%) exhibited moderate to high co-occurring symptom trajectories (Figure), with a 46% higher likelihood of worsening symptoms over time compared with children with no pain and no PSS (OR, 1.46 [95% CI, 1.33-1.61]; mean score difference, 1.91 [95% CI, 1.32-2.51]). Characteristics of the no pain trajectory groups are described in Table 1, and characteristics of the pain trajectory groups are described in Table 2. For further context and interpretation, we coded anxiety and depression symptom scores as at risk or in clinical range, based on recommended T-score cutpoints for boys and girls.^32^ Overall, 1090 children (50.8%) in the moderate to high co-occurring symptom groups combined had at-risk or clinical-range scores, compared with 507 children (5.4%) in the low to intermittent groups (OR, 17.76 [95% CI, 15.88-20.31]). Furthermore, 582 children (82.4%) in the high pain and high PSS group were classified with such scores, and compared with groups with lower pain and low PSS, children with moderate to high co-occurring pain-PSS were more likely to have persistent and worsening symptoms (OR, 2.45 [95% CI, 2.19-2.73]) and report multiregion pain (OR, 1.62 [95% CI, 1.27-2.08]).

**Figure.**
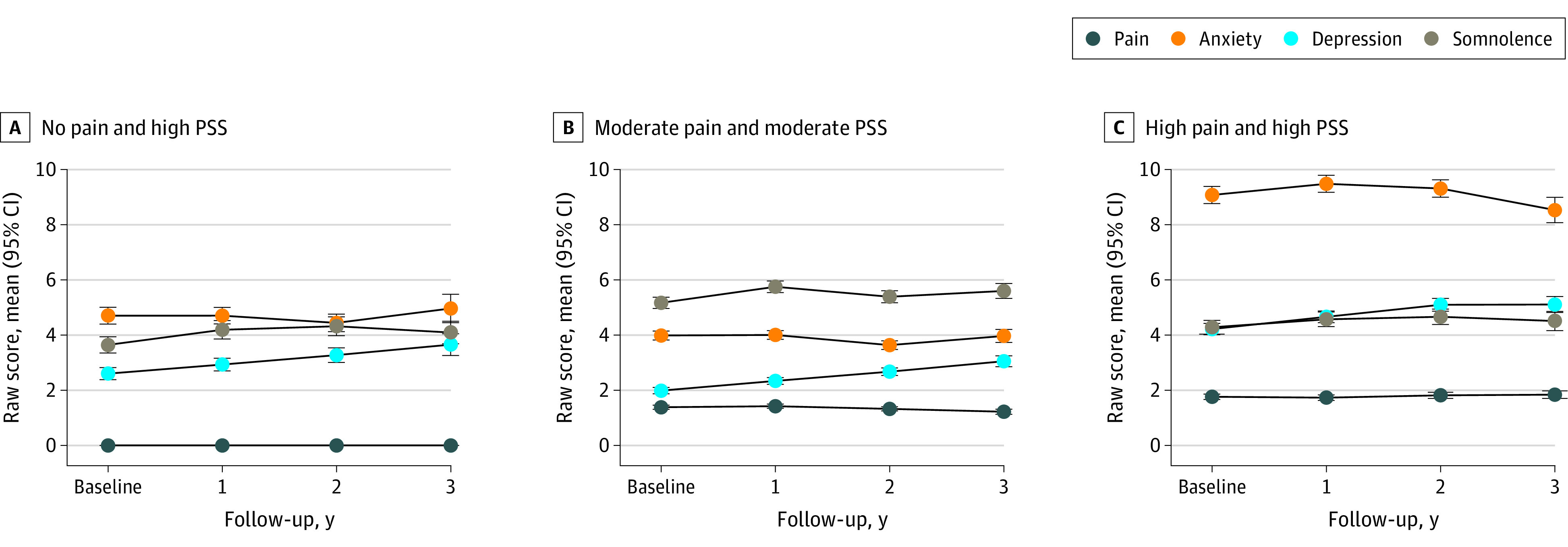
High Co-occurring Symptom Trajectories Possible range for each symptom differs: pain, 0-6; anxiety, 0-26; depression, 0-16; somnolence, 0-20. Higher scores indicate worse symptoms. PSS indicates psychological and sleep disturbance symptoms.

**Table 1.** Characteristics of the No Pain Strata Trajectory Groups

Characteristic	Children, No. (%) (n = 4031)			
	No pain and no PSS (n = 1746)	No pain and low somnolence (n = 842)	No pain and low anxiety (n = 1014)	No pain and high PSS (n = 429)
Baseline trajectory symptoms, mean (SD) (possible range)				
Anxiety (0-26)	0.41 (0.74)	0.94 (1.15)	2.85 (2.18)	4.70 (3.2)
Depression (0-16)	0.15 (0.45)	0.32 (0.63)	1.05 (1.32)	2.60 (2.29)
Somnolence (0-20)	0.38 (0.73)	2.60 (2.16)	0.88 (1.10)	3.64 (3.09)
At-risk and clinical-range anxiety or depression^a^	0	2 (<0.1)	78 (7.7)	191 (44.5)
Sex				
Male	967 (55.4)	469 (55.7)	575 (56.7)	244 (56.9)
Female	779 (44.6)	373 (44.3)	439 (43.3)	185 (43.1)
Age at final report, mean (SD), y	12.28 (0.92)	12.28 (0.96)	12.33 (0.95)	12.25 (0.97)
Puberty stage 4-5 at final report	457 (26.2)	232 (27.7)	295 (29.2)	121 (28.3)
Race				
Black	369 (21.5)	175 (21.1)	134 (13.6)	63 (15.3)
Multiracial	152 (8.9)	95 (11.5)	124 (12.5)	61 (14.3)
White	1029 (60.1)	497 (59.9)	636 (64.3)	267 (62.7)
Other^b^	163 (9.5)	63 (7.6)	95 (9.6)	33 (7.8)
Hispanic ethnicity	409 (23.7)	142 (17.1)	236 (23.5)	82 (19.3)
Household annual income <$35 000	356 (22.7)	154 (19.9)	180 (19.6)	90 (22.6)
Medical history and other symptoms at baseline (possible range)				
Comorbidity burden ≥2	187 (10.7)	118 (14.0)	121 (12.0)	79 (18.4)
Somatic symptoms, mean (SD) (0-16)	0.15 (0.44)	0.25 (0.53)	0.32 (0.63)	0.53 (0.82)
Inattention symptoms, mean (SD) (0-20)	1.07 (1.87)	2.42 (2.84)	2.77 (3.17)	5.34 (4.28)
Health care visits in the past 12 mo at the final report				
≥1 Mental health visits	70 (4.0)	60 (7.2)	110 (11.0)	133 (31.7)
≥1 Nonroutine medical visits	477 (27.3)	288 (34.2)	318 (31.4)	177 (41.3)

Abbreviation: PSS, psychological and sleep disturbance symptoms.

^a^ At-risk and clinical-level anxiety or depression were classified based on T-scores greater than 60 for boys or girls.^32^

^b^ Includes American Indian, Asian, Native Hawaiian, and other Pacific Islander.

**Table 2.** Characteristics of the Pain Strata Trajectory Groups

Characteristic	Children, No. (%) (n = 7442)				
	Low pain and no PSS (n = 2110)	Low pain and low somnolence (n = 1631)	Low pain and low anxiety (n = 1984)	Moderate pain and moderate PSS (n = 1011)	High pain and high PSS (n = 706)
Baseline trajectory symptoms, mean (SD) (possible range scores)					
Pain (0-6)	0.72 (0.79)	0.93 (0.92)	1.12 (1.04)	1.39 (1.20)	1.76 (1.33)
Anxiety (0-26)	0.79 (1.07)	1.51 (1.48)	4.00 (2.65)	3.99 (2.63)	9.07 (4.20)
Depression (0-16)	0.23 (0.54)	0.58 (0.91)	1.35 (1.58)	1.99 (1.87)	4.22 (2.76)
Somnolence (0-20)	0.70 (0.96)	2.86 (2.05)	1.35 (1.37)	5.17 (3.31)	4.28 (3.38)
At-risk and clinical-range anxiety or depression^a^	6 (0.3)	24 (1.5)	397 (20.0)	317 (31.4)	582 (82.4)
Sex					
Male	1106 (52.4)	810 (49.7)	987 (49.8)	505 (50.0)	355 (50.3)
Female	1004 (47.6)	821 (50.3)	997 (50.3)	506 (50.1)	351 (49.7)
Age at final report, mean (SD), y	12.45 (0.88)	12.49 (0.87)	12.41 (0.87)	12.52 (0.85)	12.44 (0.89)
Puberty stage 4-5 at final report	609 (29.0)	539 (33.1)	635 (32.0)	374 (37.0)	236 (33.6)
Race					
Black	352 (16.9)	250 (15.5)	186 (9.5)	143 (14.4)	70 (10.1)
Multiracial	232 (11.1)	208 (12.9)	272 (14.0)	159 (16.0)	107 (15.4)
White	1367 (65.6)	1051 (65.1)	1384 (71.0)	641 (64.4)	470 (67.5)
Other^b^	132 (6.3)	105 (6.5)	108 (5.5)	53 (5.3)	49 (7.0)
Hispanic ethnicity	394 (18.9)	277 (17.2)	403 (20.5)	204 (20.6)	153 (22.0)
Household annual income <$35 000	348 (17.8)	297 (19.7)	326 (17.5)	211 (23.2)	182 (28.4)
Medical history and other symptoms at baseline (possible range)					
Comorbidity burden ≥2	366 (17.4)	358 (22.0)	423 (21.3)	274 (27.2)	202 (28.9)
Somatic symptoms, mean (SD) (0-16)	0.41 (0.75)	0.68 (0.95)	0.90 (1.14)	1.34 (1.48)	2.10 (1.78)
Inattention symptoms, mean (SD) (0-20)	1.56 (2.21)	2.87 (2.98)	3.47 (3.42)	5.08 (3.92)	7.13 (4.51)
Child’s self-reported pain characteristics, mean (SD)^c^					
Pain intensity (0-10)	6.2 (2.2)	6.2 (2.3)	6.1 (2.2)	6.2 (2.5)	6.2 (2.3)
Pain interference (0-10)	1.9 (2.3)	1.9 (2.3)	2.1 (2.4)	2.4 (2.5)	2.5 (2.0)
Multiregion pain	212 (17.2)	218 (23.1)	247 (21.7)	140 (24.5)	113 (30.2)
Health care visits (final report)					
≥1 Mental health visits	108 (5.2)	142 (8.8)	347 (17.6)	238 (23.9)	318 (45.9)
≥1 Nonroutine medical visits	736 (34.9)	654 (40.1)	856 (43.2)	442 (43.7)	348 (49.3)

Abbreviation: PSS, psychological and sleep disturbance symptoms.

^a^ At-risk and clinical-level anxiety or depression were classified based on T-scores greater than 60 for boys or girls.^32^

^b^ Includes American Indian, Asian, Native Hawaiian, and other Pacific Islander.

^c^ Among 4262 children who self-reported past month pain in year 3. Children who reported no pain did not score pain intensity or interference; body map areas were coded as no pain, accordingly.

Symptom groups differed by sex, race, and ethnicity when controlled for puberty, comorbidity burden, and somatic and inattention symptoms (Table 3). With the asymptomatic group (no pain and no PSS) as comparator, girls were at higher risk relative to boys of having higher symptom trajectories, while children who were Black (aRRR range, 0.15-0.38), other race (aRRR range, 0.43-0.59), or Hispanic (aRRR range, 0.58-0.67) were at lower risk compared with White or non-Hispanic children (Table 3). Post hoc analysis found that girls were more likely to have worsening symptoms over time (OR, 1.17 [95% CI, 1.09-1.26]) and had higher final symptom scores (mean difference, 0.72 [95% CI, 0.4-1.0]).

**Table 3.** Association Between Child Characteristics and Group Membership

Factor	Relative risk ratio (95% CI) (n = 11 104)^a^									
	Low, intermittent, or single symptom clusters						High co-occurring symptom clusters			
	No pain and low somnolence	No pain and low anxiety	Low pain and no PSS	Low pain and low somnolence	Low pain and low anxiety	No pain and high PSS		Moderate pain and moderate PSS	High pain and high PSS
Female sex^b^	1.21 (0.96-1.52)	1.16 (0.93-1.44)	1.19 (1.00-1.43)	1.35 (1.11-1.65)	1.61 (1.33-1.95)	1.53 (1.14-2.05)		1.73 (1.37-2.19)	2.41 (1.84-3.15)
Puberty status									
Prepuberty or early	1 [Reference]	1 [Reference]	1 [Reference]	1 [Reference]	1 [Reference]	1 [Reference]		1 [Reference]	1 [Reference]
Middle	0.99 (0.79-1.24)	1.02 (0.82-1.27)	1.11 (0.93-1.33)	1.49 (1.22-1.83)	1.20 (0.99-1.46)	1.35 (1.01-1.81)		1.24 (0.97-1.58)	1.21 (0.91-1.59)
Late	1.21 (0.91-1.61)	1.37 (1.05-1.81)	1.29 (1.02-1.62)	2.00 (1.55-2.57)	1.58 (1.24-2.02)	1.69 (1.18-2.43)		2.12 (1.59-2.84)	1.71 (1.22-2.38)
Race									
Black	0.79 (0.61-1.02)	0.46 (0.35-0.60)	0.62 (0.50-0.76)	0.44 (0.35-0.56)	0.22 (0.17-0.29)	0.38 (0.26-0.53)		0.31 (0.23-0.41)	0.15 (0.10-0.21)
Multiracial	1.27 (0.93-1.74)	1.24 (0.92-1.66)	1.04 (0.81-1.35)	1.13 (0.86-1.48)	1.05 (0.81-1.36)	1.16 (0.80-1.68)		1.20 (0.89-1.61)	0.98 (0.70,1.38)
White	1 [Reference]	1 [Reference]	1 [Reference]	1 [Reference]	1 [Reference]	1 [Reference]		1 [Reference]	1 [Reference]
Other^c^	0.93 (0.64-1.35)	0.91 (0.65-1.27)	0.61 (0.45-0.82)	0.60 (0.43-0.84)	0.45 (0.33-0.63)	0.82 (0.52-1.30)		0.43 (0.28-0.65)	0.59 (0.38-0.92)
Hispanic ethnicity^d^	0.59 (0.45-0.77)	0.81 (0.64-1.04)	0.72 (0.58-0.88)	0.59 (0.46-0.74)	0.67 (0.54-0.83)	0.66 (0.48-0.92)		0.67 (0.51-0.87)	0.58 (0.43-0.78)
Comorbidity burden ≥2^e^	1.33 (1.01-1.77)	1.06 (0.80-1.40)	1.74 (1.40-2.17)	2.14 (1.70-2.69)	2.07 (1.65-2.60)	1.76 (1.27-2.43)		2.37 (1.83-3.06)	2.29 (1.72-3.05)
Somatic symptoms	1.46 (1.20-1.77)	1.77 (1.49-2.11)	2.31 (1.99-2.70)	3.19 (2.74-3.72)	3.85 (3.31-4.47)	2.34 (1.94-2.83)		4.64 (3.98-5.41)	5.92 (5.06-6.93)
Inattention symptoms	1.38 (1.32-1.44)	1.43 (1.37-1.49)	1.16 (1.12-2.21)	1.43 (1.38-1.50)	1.51 (1.45-1.58)	1.75 (1.67-1.83)		1.67 (1.60-1.75)	1.85 (1.77-1.94)

Abbreviation: PSS, psychological and sleep disturbance symptoms.

^a^ Results from mixed effect multinomial regression including all trajectory groups, with the asymptomatic group used as the reference group; all covariates included in the analysis are depicted in the first column, with family and research site included as random intercepts. Model statistics: likelihood ratio χ^2^_2_(fixed nested within random) = 30.97; *P* < .001.

^b^ Reference: male.

^c^ Includes American Indian, Asian, Native Hawaiian, and other Pacific Islander.

^d^ Reference: non-Hispanic.

^e^ Reference: less than 2.

Our second hypothesis was supported by a graduated increase in the likelihood of nonroutine HCU across symptom trajectories (Table 4). Children in the highest co-occurring symptom trajectories were 13 to 27 times more likely than the asymptomatic group to have utilized mental health care services, adjusting for confounding (no pain and high PSS: aOR, 13.07 [95% CI, 8.74-19.53]; high pain and high PSS: aOR, 26.84 [95% CI, 17.89-20.29]). Furthermore, the high pain and high PSS group had significantly greater rates of HCU compared with every other trajectory (eg, aOR vs asymptomatic group, 2.43 [95% CI, 1.97-2.99]) (Table 4). Importantly, despite their relatively low utilization overall, children with at-risk or clinical-range anxiety or depression scores were 5 times more likely to use mental health services (OR, 6.16 [95% CI, 5.45-6.96]) and nonroutine medical care (OR, 1.58 [95% CI, 1.42-1.75]).

**Table 4.** Association Between Symptom Trajectory Group and Final Year Health Care Utilization

Fixed factors	Adjusted odds ratio (95% CI)^a^	
	Nonroutine medical visit (n = 10 219)	Mental health care (n = 10 122)
Trajectory group		
Asymptomatic	1 [Reference]	1 [Reference]
No pain and low somnolence	1.36 (1.11-1.66)	1.87 (1.24-2.83)
No pain and low anxiety	1.19 (0.98-1.44)	3.04 (2.10-4.40)
Low pain and no PSS	1.37 (1.17-1.60)	1.33 (0.93-1.89)
Low pain and low somnolence	1.71 (1.45-2.02)	2.38 (1.69-3.37)
Low pain and low anxiety	1.88 (1.61-2.21)	5.65 (4.08-7.81)
No pain and high PSS	1.73 (1.36-2.20)	13.07 (8.74-19.53)
Moderate pain and moderate PSS	1.94 (1.61-2.35)	8.59 (6.04-12.22)
High pain and high PSS	2.43 (1.97-2.99)	26.84 (17.89-20.29)
Female sex^b^	1.07 (0.97-1.19)	0.89 (0.76-1.05)
Puberty status		
Prepuberty or early	1 [Reference]	1 [Reference]
Middle	0.95 (0.85-1.06)	0.97 (0.82-1.15)
Late	0.87 (0.76-0.99)	1.09 (0.89-1.34)
Race		
Black race	0.61 (0.52-0.71)	0.68 (0.54-0.87)
Multiracial	0.76 (0.66-0.87)	1.01 (0.82-1.24)
White	1 [Reference]	1 [Reference]
Other^c^	0.72 (0.59-0.87)	0.68 (0.48-0.95)
Hispanic ethnicity^d^	0.95 (0.83-1.08)	0.59 (0.47-0.73)
Comorbidity burden ≥2^e^	1.52 (1.36-1.70)	1.26 (1.07-1.49)
Household annual income ≤$35 000^f^	0.87 (0.77-0.99)	1.10 (0.91-1.33)
Wald χ^2^		
Estimate (*df*)	253.96 (17)	432.99 (17)
*P* value	<.001	<.001

Abbreviation: PSS, psychological and sleep disturbance symptoms.

^a^ Data derived from Generalized Mixed Effects Logistic Regression models with family and site included as random intercepts.

^b^ Reference: male.

^c^ Includes American Indian, Asian, Native Hawaiian, and other Pacific Islander.

^d^ Reference: non-Hispanic.

^e^ Reference: less than 2.

^f^ Reference: annual income more than $35 000.

Nonroutine HCU differed by race, ethnicity, and household income (Table 4). Furthermore, in the subgroup with highest co-occurring symptom trajectories (ie, no pain and high PSS, moderate pain and moderate PSS, and high pain and high PSS groups combined), Black children were less likely to have nonroutine medical care (OR, 0.57 [95% CI, 0.43-0.76]) or mental health care (OR, 0.58 [95% CI, 0.42-0.81]) visits compared with White children, even among those with at-risk and clinical-range scores (eTable 2 in Supplement 1). Hispanic children were less likely to have received mental health care services compared with non-Hispanic children (OR, 0.67 [95% CI, 0.48-0.90]), but they were similar to non-Hispanic children in reporting nonroutine medical care visits (OR, 0.89 [95% CI, 0.67-1.18]). These racial and ethnic differences remained when contrasted by low- and high-income households.

## Discussion

The findings of this cohort study present a contemporary view of co-occurring symptom trajectories in a large US sample of relatively healthy children. On a positive note, most of the cohort (81%) had asymptomatic or low, intermittent, or single symptom trajectories. However, nearly 1 in 5 children had moderate to high co-occurring symptom trajectories that persisted or worsened for most. These rates align with reports of single symptom trajectories during adolescence suggesting concerning rates of persistent, recurrent, or worsening pain,^6,8,10,18^ anxiety and depression,^9,41^ and sleep disturbance symptoms.^13,42^ Notably, fewer than half of the children with moderate to high co-occurring symptom trajectories reported nonroutine HCU, even when scores reached at-risk or clinical levels. Furthermore, racially minoritized children, such as Black children, and those from lower income households were least likely to report nonroutine HCU, even when faced with moderate to high co-occurring symptom trajectories or at-risk or clinical-range depression or anxiety scores. The implications of these findings are significant, suggesting that routine primary care may offer the only opportunity for children at highest risk to have their co-occurring symptoms addressed before they worsen or become persistent.

Our study extends previous findings by showing how pain, psychological, and sleep disturbance symptoms may evolve and worsen simultaneously over time for many youths. Conflicting data support temporal directions from internalizing symptoms toward increasing or persistent pain,^8,10,17^ from sleep disturbance toward persistent pain and mental health symptoms,^43,44,45^ and the reverse, from chronic pain to internalizing symptoms.^17,19,46^ In contrast, our findings suggest concurrent emergence of symptoms for many children. Risk for comorbid symptoms may have genetic,^18^ neurodevelopmental,^47^ environmental,^21^ or mutual maintenance or reinforcing^46^ mechanisms. Further study incorporating a broader array of biologic and environmental factors is warranted to reveal potential mechanisms for symptom risk during this developmental period.

Importantly, girls were at greater risk than boys, while Black, other race, and Hispanic children were less likely to have moderate to high co-occurring symptom trajectories. A 2015 study among young adolescents found no sex differences in trajectories but higher anxiety and depression symptoms for girls.^41^ Conflicting findings may, in part, be attributed to pubertal development, since sex differences in pain and internalizing symptoms likely emerge in late puberty or after puberty, as our analysis suggests. Our findings regarding racial differences in symptoms differ from those from a large sample of urban-based middle school students, in which Black boys reported greater anxiety and Hispanic girls, higher depression symptoms, compared with other racial and ethnic groups.^48^ Furthermore, a 2009 study by Adkins et al^49^ found that compared with White youths, non-White youths had higher depressive symptom trajectories from middle school into young adulthood, an association modified by lower socioeconomic status and stressful life events in another national sample. Our divergent findings may be attributed to the inclusion of multiple co-occurring symptoms, stratification by pain reports, and use of parent-reported symptoms. Indeed, despite moderate parent-child concordance for presence and frequency of somatic symptoms, particularly in children with pain diagnoses,^50^ lower concordance has been demonstrated among mostly Black, urban families, in which children tended to report higher symptoms than their parents.^51^ Concordance is greater for children with higher symptom burdens and those with headache and abdominal, back, and neck pain, but lowest for other (perhaps less visible) pains, anxiety, and depression symptoms,^50,52^ suggesting more reports by children or better observation by parents as symptoms worsen. Our finding of more self-reported multiregion pain among children with higher symptom trajectories (based on parent-report) supports this notion. However, our findings should be interpreted with caution, since many symptom scores did not reach at-risk or clinical levels, leaving the potential for underestimation or overestimation by parents.

Despite a higher likelihood of nonroutine HCU among children with the highest comorbid symptom trajectories and in those with at-risk or clinical-range scores, less than half reported such visits. Similarly, half of US youths with treatable mental health symptoms in 2016 did not receive treatment.^53^ Since most mental health services for symptomatic youths are received in outpatient or school settings,^54^ COVID-19 pandemic–related closures could have reduced children’s access during the ABCD longitudinal assessments. Furthermore, many of the anxiety and depression symptom scores did not reflect at-risk or clinical levels (eg, 14% overall had at-risk or clinical-range scores), and parents may have believed symptoms to be manageable at home or unworthy of a health care visit during the pandemic. That racially minoritized children and those in lower income households were less likely to use nonroutine health care is concerning but not surprising, given ongoing inequities in primary and specialty care HCU among US children.^55^ Similar to our findings, a 2016 study by Marrast et al^56^ found that mental health care disparities persist for Black and Hispanic children even when controlled for household income and other factors.^56^ Hypothesized reasons for such disparities include structural inequities, like lack of minority health care practitioners or residence in health care deserts, as well as multigenerational medical distrust and cautious preference for informal sources of counseling, like family or religious leaders.^56,57^ Further exploration of individual- and community-level contexts for disparities is warranted, given associations between mental health workforce shortage areas and negative outcomes in children.^58^ This is particularly important to guide policy and innovative strategies to address physical and mental health symptoms for youths at the most risk.

### Limitations

This study has some limitations. Although the ABCD sample is diverse, children from rural areas and lower-income households are underrepresented, limiting the ability to generalize findings.^26^ Also, since study participation is voluntary, the potential for selection bias exists, with nonparticipants differing in unknown ways. Attrition has been higher among participants with Spanish-speaking parents,^29^ jeopardizing interpretation of ethnicity-associated findings. Furthermore, whether pandemic-associated biological, social, and environmental factors impacted participants’ symptoms is unknown but worthy of exploration, given reports of new-onset chronic pain, preceded by higher depression and anxiety in children during this era.^59^ Additionally, ABCD surveys were conducted virtually from 2020 to 2022, which may have influenced responses in unknown ways.^60^

## Conclusion

This cohort study found that approximately 1 in 5 children presented with moderate to high co-occurring symptom trajectories that persisted or worsened from age 9 to 13 years, suggesting emergence of a phenotypic symptom profile during early adolescence. This is alarming, given findings that half of boys and two-thirds of girls with 1 or more episodes of high mental health symptoms during adolescence had persistent symptoms into adulthood.^9^ Since single-symptom episodes of short duration are less likely to persist,^9^ early recognition and prompt intervention is essential. Our findings of low and disparate HCU among youths at highest risk for symptom persistence has important implications for primary care practitioners, suggesting a need for early recognition and comprehensive management.

## Notes

Supplement 1. **eTable 1.** Trajectory Model Solution Statistics With Posterior Probabilities by Group **eTable 2.** Subgroup Analyses of Health Care Utilization by Race and Ethnicity for Children in the Highest Co-occurring Symptom Trajectories **eFigure.** Low, Intermittent, or Single Symptom Trajectories

Supplement 2.
Data Sharing Statement
