# Supplementary material for: Associations of Co-occurring Symptom Trajectories With Sex, Race, Ethnicity, and Health Care Utilization in Children
Source: JAMA Netw Open. 2023 May 18;6(5):e2314135. doi: 10.1001/jamanetworkopen.2023.14135 (PMC10196876; doi:10.1001/jamanetworkopen.2023.14135)
Supplement: Supplement 1. — eTable 1. Trajectory Model Solution Statistics With Posterior Probabilities by Group eTable 2. Subgroup Analyses of Health Care Utilization by Race and Ethnicity for Children in the Highest Co-occurring Symptom Trajectories eFigure. Low, Intermittent, or Single Symptom Trajectories [file jamanetwopen-e2314135-s001.pdf]

Supplemental Online Content

Voepel-Lewis T, Senger-Carpenter T, Chen B, et al. Associations of co-occurring symptom trajectories with sex, race, ethnicity, and health care utilization in children. *JAMA Netw Open*. 2023;6(5):e2314135. doi:10.1001/jamanetworkopen.2023.14135

eTable 1. Trajectory Model Solution Statistics With Posterior Probabilities by Group

eTable 2. Subgroup Analyses of Health Care Utilization by Race and Ethnicity for Children in the Highest Cooccurring Symptom Trajectories

eFigure. Low, Intermittent, or Single Symptom Trajectories

This supplemental material has been provided by the authors to give readers additional information about their work.

© 2023 Voepel-Lewis T et al. *JAMA Network Open.*

eTable 1. Trajectory Model Solution Statistics With Posterior Probabilities by Group

Solutions	Membership n (%) based on max likelihood rule	Posterior Probabilities Average (SD); range	% Members with PP> 0.7; 0.8; 0.9	Log likelihood	BIC	Entropy
**Pain group solutions (n=7442)**						
**4 groups**		**.90 to .96**		**-180784.09**	**-181039.09**	**0.871**
Group 1	2,796 (37.57)	.96 (.11); .40-1.00				
Group 2	1,511 (20.30)	.90 (.15); .37-1.00				
Group 3	2,069 (27.80)	.90 (.15); .36-1.00				
Group 4	1,066 (14.32)	.96 (.10); .43-1.00				
**5 groups**		**.87 to .96**		**-178945.76**	**-179240.17**	**0.852**
Group 1	2,110 (28.4)	.93 (.13); .37-.99	91; 84.6; 77.5			
Group 2	1,631 (21.9)	.87 (.16); .38-.99	80.8; 71.7; 60.1			
Group 3	1,011 (13.59)	.90 (.15); .42-1.00	86.5; 78.2; 68.6			
Group 4	1,984 (26.7)	.91 (.14); .36-1.00	87.7; 80.6; 71.8			
Group 5	706 (9.49)	.96 (.10); .38-1.00	94.8; 91.5; 87.1			
**6 groups**		**.86 to .95**		**-177223.73**	**-177512.36**	**0.846**
Group 1	1,741 (23.4)	.91 (.14); .39-.99				
Group 2	1,330 (17.9)	.86 (.16); .35-.99				
Group 3	2,018 (27.1)	.88 (.16); .35-.99				
Group 4	1,038 (14.0)	.89 (.15); .40-.99				
Group 5	934 (12.6)	.90 (.14); .39-.99				
Group 6	381 (5.1)	.95 (.11); .42-1.00				
**No Pain Group Solutions (n=4031)**						
**3 groups**		**.92 to .94**		**-59293.47**	**-59420.70**	**0.855**
Group 1	1,802 (44.7)	.94 (.11); .50-.99				
Group 2	1,664 (41.3)	.92 (.13); .50-.99				
Group 3	565 (14.0)	.94 (.11); .50-1.00				
**4 groups**		**.90 to .95**		**-57595.16**	**-57754.20**	**0.855**
Group 1	1,746 (43.3)	.93 (.13); .38-.99	91.5; 87.5; 80.1			
Group 2	842 (20.9)	.90 (.14); .41-1.00	85.6; 79.2; 66.8			
Group 3	1,014 (25.2)	.91 (.14); .36-.99	87.5; 82.2; 71.3			
Group 4	429 (10.6)	.95 (.11); .46-1.00	93.7; 90.0; 83.7			
**5 groups**		**.87 to .93**		**-56896.70**	**-57098.15**	**0.837**
Group 1	1,252 (31.1)	.91 (.14); .42-.99				
Group 2	1,111 (27.6)	.87 (.16); .35-.99				
Group 3	1,009 (25.0)	.91 (.14); .41-.99				
Group 4	359 (8.9)	.89 (.15); .41-.99				
Group 5	300 (7.4)	.93 (.13); .39-1.00				

SD=Standard Deviation; PP=posterior probabilities; BIC=Bayesian Information Criterion

© 2023 Voepel-Lewis T et al. *JAMA Network Open.*

eTable 2. Subgroup Analyses of Health Care Utilization by Race and Ethnicity for Children in the Highest Cooccurring Symptom Trajectories

	**Unplanned Medical Visit**	Odds Ratio [95% CI][a]	**Mental Health Service**	Odds Ratio [95% CI][a]
White race (n=1293)	623 (48.2)		456 (35.8)	
High income (n=1087)	525 (48.3)		383 (35.7)	
Low income (n=206)	98 (47.6)		73 (36.3)	
At risk/clinical range (n=706)	361 (50.4)		335 (47.5)	
Black race (n=234)	81 (34.6)	0.57 [0.43, 0.76] [b]	56 (24.6)	0.58 [0.42, 0.81] [b]
High income (n=89)	27 (30.3)	0.47 [0.29, 0.74] [c]	21 (24.4)	0.58 [0.35, 0.97] [c]
Low income (n=145)	54 (37.2)	0.65 [0.42, 1.01] [d]	35 (24.6)	0.57 [0.36, 0.93] [d]
At risk/clinical range (n=114)	44 (38.6)	0.62 [0.41, 0.93] [e]	36 (32.7)	0.54 [0.35, 0.82] [e]
Non-Hispanic (n=1545)	713 (46.1)		447 (35.4)	
High income (n=1220)	576 (47.2)		428 (35.6)	
Low income (n=325)	137 (42.2)		108 (34.7)	
At risk/clinical range (n=834)	412 (49.4)		389 (47.8)	
Hispanic (n=373)	155 (41.6)	0.83 [0.66, 1.04] [f]	59 (26.7)	0.60 [0.47, 0.78] [f]
High income (n=227)	88 (38.8)	0.71 [0.53, 0.95] [g]	57 (25.3)	0.61 [0.44, 0.85] [g]
Low income (n=146)	67 (45.9)	1.16 [0.79, 1.72] [h]	35 (24.1)	0.60 [0.38, 0.93] [h]
At risk/clinical range (n=238)	100 (42.0)	0.74 [0.56, 0.99] [i]	77 (32.4)	0.52 [0.39, 0.71] [i]

Data from the combined trajectories No Pain/High Psychologic, Sleep disturbance Symptoms (PSS), Moderate Pain/Moderate PSS, High Pain/High PSS.

[a]Odds ratio [95% confidence interval] represent unadjusted, univariate comparisons (Chi-square tests) with comparator groups as follows: [b]White, [c]White High Income, [d]White Low Income, [e]White at risk/clinical, [f]Non-Hispanic, [g] Non-Hispanic High Income, [h]Non-Hispanic Low Income, [i]Non-Hispanic at risk

© 2023 Voepel-Lewis T et al. *JAMA Network Open.*

eFigure 1. Low, Intermittent, or Single Symptom Trajectories

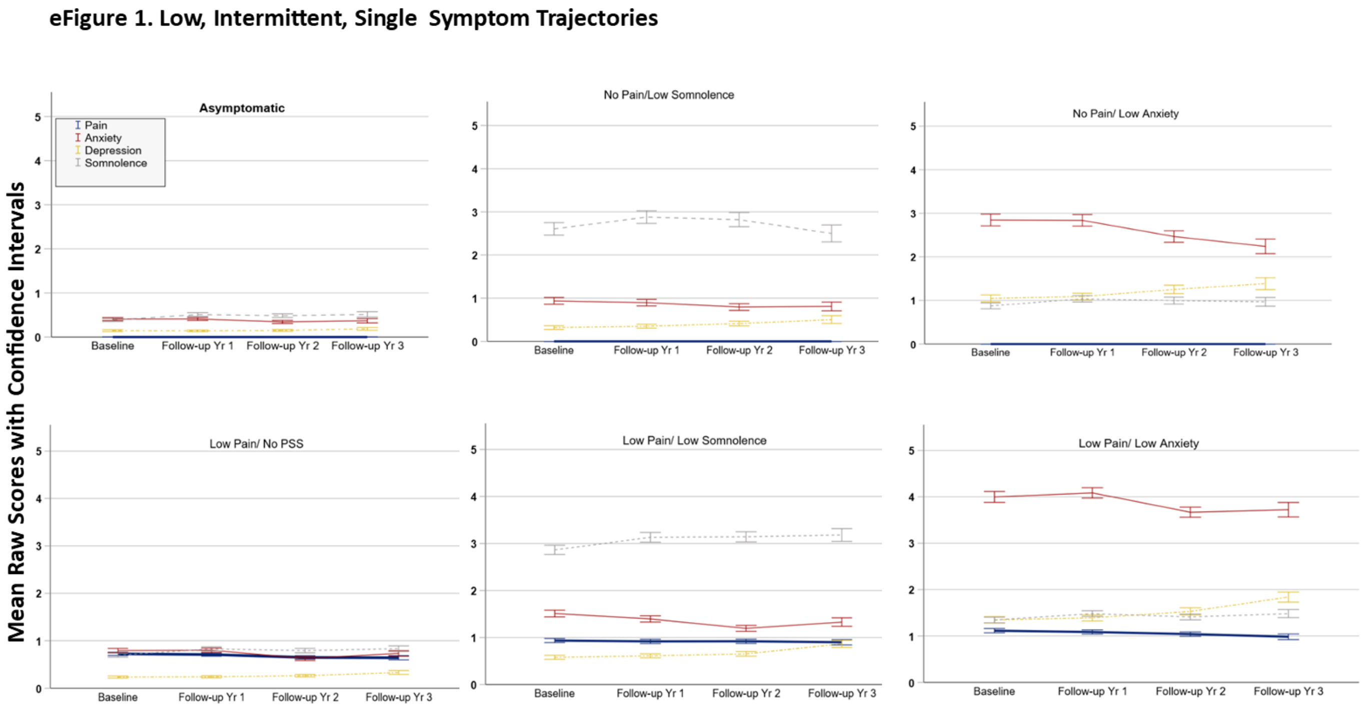

Mean raw symptom scores with 95% Confidence Intervals (CI) are depicted from Baseline through follow-up year 3. Scale in figure is restricted to range of 0-5 to best depict low score changes over time; Possible range for each symptom differs (i.e., Pain 0-6, Anxiety 0-26, Depression 0-16, Somnolence 0-20). PSS = Psychological and Sleep disturbance Symptoms.

© 2023 Voepel-Lewis T et al. *JAMA Network Open.*
