# Supplementary material for: Associations of Co-occurring Symptom Trajectories With Sex, Race, Ethnicity, and Health Care Utilization in Children
Source: JAMA Netw Open. 2023 May 18;6(5):e2314135. doi: 10.1001/jamanetworkopen.2023.14135 (PMC10196876; doi:10.1001/jamanetworkopen.2023.14135)
Supplement: Supplement 2. — Data Sharing Statement [file jamanetwopen-e2314135-s002.pdf]

Data Sharing Statement

Voepel-Lewis. Associations of Co-occurring Symptom Trajectories With Sex, Race, Ethnicity, and Health Care Utilization in Children. *JAMA Netw Open*. Published May 18, 2023. doi:10.1001/jamanetworkopen.2023.14135

Data
Data available: Yes
Data types: Deidentified participant data
How to access data: URL to be determined by ABCD; all data are uploaded to the ABCD (NIMH data archive) per our data use agreement with ABCD
When available: With publication

Supporting Documents
Document types: None

Additional Information
Who can access the data: Researchers who are granted a DUA from NIMH for use of ABCD datasets
Types of analyses: must be used for specified secondary analyses through DUA with NIMH/ABCD study coordinators
Mechanisms of data availability: With DUA only
Any additional restrictions: Per NIMH data use agreement
